# Shank Muscle Strength Training Changes Foot Behaviour during a Sudden Ankle Supination

**DOI:** 10.1371/journal.pone.0130290

**Published:** 2015-06-25

**Authors:** Marco Hagen, Stephanie Lescher, Andreas Gerhardt, Matthias Lahner, Stephan Felber, Ewald M. Hennig

**Affiliations:** 1 Biomechanics Laboratory, Institute of Sport and Movement Sciences, University Duisburg Essen, Essen, Germany; 2 Institute of Neuroradiology, University of Frankfurt, Frankfurt, Germany; 3 Department of Orthopaedic Sports Surgery, St. Josef-Hospital, Ruhr-University Bochum, Bochum, Germany; 4 Institute for Diagnostic and Interventional Radiology, Neuroradiology and Nuclear Medicine, Stiftungsklinikum Mittelrhein Koblenz, Koblenz, Germany; Universidad Europea de Madrid, SPAIN

## Abstract

**Background:**

The peroneal muscles are the most effective lateral stabilisers whose tension braces the ankle joint complex against excessive supination. The purpose of this study was to identify the morphological and biomechanical effects of two machine-based shank muscle training methods.

**Methods:**

Twenty-two healthy male recreationally active sports students performed ten weeks of single-set high resistance strength training with 3 training sessions per week. The subjects conducted subtalar pronator/supinator muscle training (ST) with the right leg by using a custom-made apparatus; the left foot muscles were exercised with machine-based talocrural plantar and dorsiflexor training (TT). Muscle strength (MVIC), muscle volume and foot biomechanics (rearfoot motion, ground reaction forces, muscle reaction times) during a sudden ankle supination were recorded before and after the intervention.

**Results:**

Compared to TT, ST resulted in significantly higher pronator (14% vs. 8%, P<0.01) and supinator MVIC (25% vs. 12%, P<0.01). During sudden foot inversions, both ST and TT resulted in reduced supination velocity (-12%; P<0.01). The muscle reaction onset time was faster after the training in peroneus longus (PL) (P<0.01). Muscle volume of PL (P<0.01) and TA (P<0.01) increased significantly after both ST and TT.

**Conclusion:**

After both ST and TT, the ankle joint complex is mechanically more stabilised against sudden supinations due to the muscle volume increase of PL and TA. As the reduced supination velocities indicate, the strength training effects are already present during free-fall. According to a sudden ankle supination in standing position, both machine-based dorsiflexor and pronator strength training is recommended for enhancing the mechanical stability of the ankle.

## Introduction

Strength training is frequently applied in the prevention of sports-related injuries, mostly with the intention to enhance joint stability. The increase of mechanical joint stiffness by stronger bracing of the bony joint structures due to strength training depends on, amongst other changes, to what extent the volume of the stabilizing muscles can be enhanced [1]. Consequently, a major goal of preventive strength training is muscle hypertrophy.

The most frequently injured anatomical region is the ankle joint complex. The ankle joint is involved in approximately every fifth sports injury [2]. A recent meta-analysis shows that the sport category with the highest incidence of ankle sprain is indoor/court sports, with a cumulative incidence rate of 7 per 1,000 exposures [3]. Chronic ankle instability (CAI) is the main residual complication following acute ankle sprain [4]. As suggested by Hertel [5], strength deficits of the muscles controlling the ankle joint complex are one functional insufficiency contributing to CAI. Based on the findings of a meta-analysis, Arnold et al. [6] assess pronator strength training as an important part of return-to-play criteria and the rehabilitation of CAI. The typical mechanism of ankle sprain injury is characterised by the transition from an unloaded to a loaded state, for instance during a landing manoeuvre [7]. As the feedback control in terms of the motor response and the electromechanical delay is too slow to protect the ankle in case of a sudden inversion [8], feedforward control by pre-activation of the peroneal muscles is most important for injury prevention. Pronator strength training may improve ankle joint neuromuscular control [9]. While neural strength training adaptations of the peroneal muscles are considered to affect the preactivation prior touchdown [7], hypertrophy increases the stiffness properties of the muscle-tendon complex [10]. Both neural and morphological adaptations are discussed to stiffen the ankle joint complex and place the foot in a more pronated position immediately prior touchdown, the position which would be the most effective strategy for preventing an ankle sprain [11].

Strength training interventions in patients with functional ankle instability are predominantly based on elastic-resistance training [12], [13], [14]. Exercising with elastic bands or tubes allows three-dimensional movement patterns and may induce neural gains [15]. As discussed by Mattacola and Dwyer [9], elastic tubes can be helpful in the early stage of rehabilitation but its use will be insufficient if morphological adaptations are aspired. For achieving muscle hypertrophy, the target muscles are not sufficiently overloaded when performing tubing exercises. This could also explain the inconsistent findings concerning muscle strength and proprioception after shank muscle strength training interventions in subjects with CAI even though the same “Thera band” training regimen was applied [12], [14]. However, this point is speculative because muscle morphology was not measured in the above-mentioned studies. To achieve gains in muscle strength as well as muscle mass, machine-based strength training is advantageous [16]. The intensity and the progression of resistance can be easily adjusted to the individual level of performance so that the fibres of the target muscles can be effectively overloaded. Furthermore, the muscle-specific angle-torque-relationship can be taken into account by using variable cams, for instance.

Another reason for the missing evidence of the stabilising effect of shank muscle training might also be the unique anatomy of the ankle joint complex. Each shank muscle crosses both the ankle and the subtalar joint axis, and acts on the one hand as a plantar or dorsiflexor and on the other hand as a pronator or supinator. The peroneal muscles are the most effective muscular lateral stabilisers of the foot [17]. Their tendons use the lateral malleolus as a hypomochlion which results in an approximately twofold moment arm to the subtalar compared to the talocrural joint axis [18]. Therefore, it can be assumed that the peroneal muscles will be most effectively strengthened if they are functionally loaded by subtalar joint-specific pronator resistance exercises. We developed a strength training machine for the pronators and supinators which was equipped with an adjustable weight block and a variable cam. The machine axis was aligned with the anatomical axis of the subtalar joint as defined by Isman and Inman [19]. Purpose of this study was to identify the morphological and biomechanical effects on the lateral stability of the foot after two machine-based shank muscle strength training methods: a) pronator and supinator strength training around the subtalar joint axis [19] (subtalar strength training = ST) compared to b) traditional plantar and dorsiflexor resistance exercising around the talocrural joint axis (talocrural strength training = TT). We hypothesised in particular, that machine-based subtalar joint-specific pronator training will increase the strength and thickness of the peroneal muscles and result in enhanced neuromuscular control of the foot during a sudden supination.

## Methods

### Study Design

The study was designed as an intervention study to investigate the biomechanical effects of ST compared to TT. Before and after the training period, every subject had to undergo muscle strength and muscle volume measurements. By using a tilting platform, a sudden ankle supination was induced and neuromuscular reactions were observed.

Training-induced increase of muscle size and strength is highly variable between individuals [20], which is related to various polymorphisms at genetic loci [21]. In a study with a small sample size, the problem of high and low responders impedes the outcome measures when comparing two groups due to the heterogeneous adaptation of subjects. Therefore, we analysed the specific effects after training the shank muscles in two different movement planes intra-individually: All subjects conducted ST with the right leg and TT with the left. Major goal of the strength training intervention was to increase muscle volume. Although we know that unilateral resistance exercise increases contralateral strength due to neural effects [22], we conducted the intra-individual comparison because morphological changes could only be observed in the trained leg [23]. Therefore, we expected to observe the hypothesised biomechanical changes as a consequence of differences in muscle hypertrophy because morphological adaptations to exercise are regulated locally [24]. For data analysis, time (pre vs. post) served as dependent variable and treatment (ST vs. TT) as independent variable. Training-specific effects are indicated by time x treatment interactions.

### Participants

Twenty-two healthy male sports students (age: 27.3±2.5 years; height: 1.83±0.07 m; body mass: 81.5±11.0 kg) were recruited for this study. All subjects have been without lower leg injuries for the last two years. During the ten-week training period, the subjects continued their usual physical exercise regimens and their university courses of physical education similar to what they were accustomed to before this experiment. Exclusion criterion for participation in this study was lower extremity strength training. All subjects were active in amateur team sports (soccer: 10, handball: 4, volleyball: 1, basketball: 1), recreational swimming (2) and/or fitness training (4). In twenty participants the right leg was dominant, in two the left leg. Leg dominance was determined as the leg which is preferred for kicking a ball. The subjects were briefed to avoid any strenuous exercise the day before the testing sessions. Approximately one week before the experiment started, the participants were instructed about the setup. After verbal explanation and a practical demonstration, the participants performed a number of practice tests.

Background information on the experimental procedures was provided to the participants, and written informed content was collected prior the first test session. The study was approved by the ethics committee of the University Hospital of Duisburg-Essen in accordance with the Helsinki Declaration.

### Instrumentation

#### Subtalar pronator/supinator strength training machine (SPSM) and strength measurements

**Fig 1:**
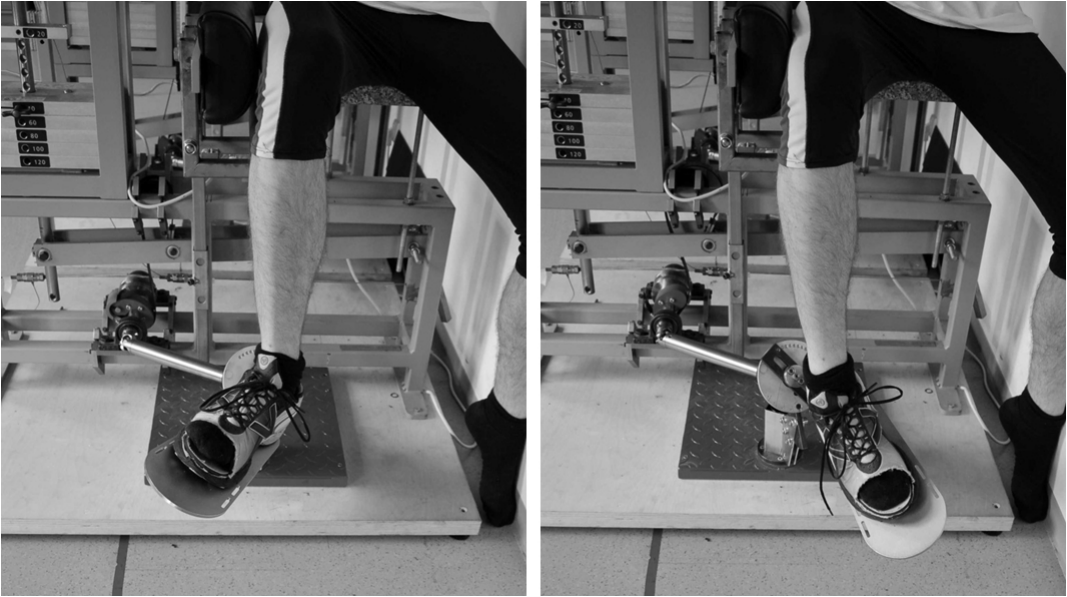
Functional strength training of pronators (left) and supinators (right) at the custom-made functional pronator/supinator strength training machine.

For performing ST, a custom-made subtalar pronator/supinator training machine (SPSM) was developed (Fig 1) [25] whose movement axis was aligned with the subtalar joint axis, as reported by Isman & Inman [19]. The foot plate was connected via a driven cardan shaft and a pull rope to the adjustable weight block, and a sports shoe (size: US 10) was mounted onto the foot plate. The tip of the shoe’s upper was removed so that work-out on the machine was possible for subjects with variable foot lengths from 26.5 to 29.5 cm (i.e. shoe sizes US 8.5 to 11.5). During the exercises, the forefoot was additionally fixed with a belt. To reduce the mechanical influence of gastrocnemius muscle, ST was performed in a seated position so that hip and knee joint each were positioned in approximately 90 degrees. During the strength training intervention, ST was conducted by using two SPSMs (one pronation machine, one supination machine). Both machines were equipped with specific variable cams [16] whose shapes had been determined in a previous experiment [25].

Before and after the intervention, each subject performed at least three valid trials of maximum voluntary isometric pronator and supinator contraction (MVIC) in a neutral position of the foot (shank perpendicular to foot sole). Trials that deviated from the shape of a ramp contraction were repeated. To reduce the error of measurement, supplementary visual biofeedback was given by displaying the signal of the corresponding force transducer on a monitor. MVIC was measured by using two force transducers (Kistler 9321A, Winterthur, CH) which were placed into a steel wire construction between the driven shaft and the frame of the SPSM. A two-minute rest was required between the trials to prevent fatigue built up [26].

#### Sudden ankle supination

**Fig 2:**
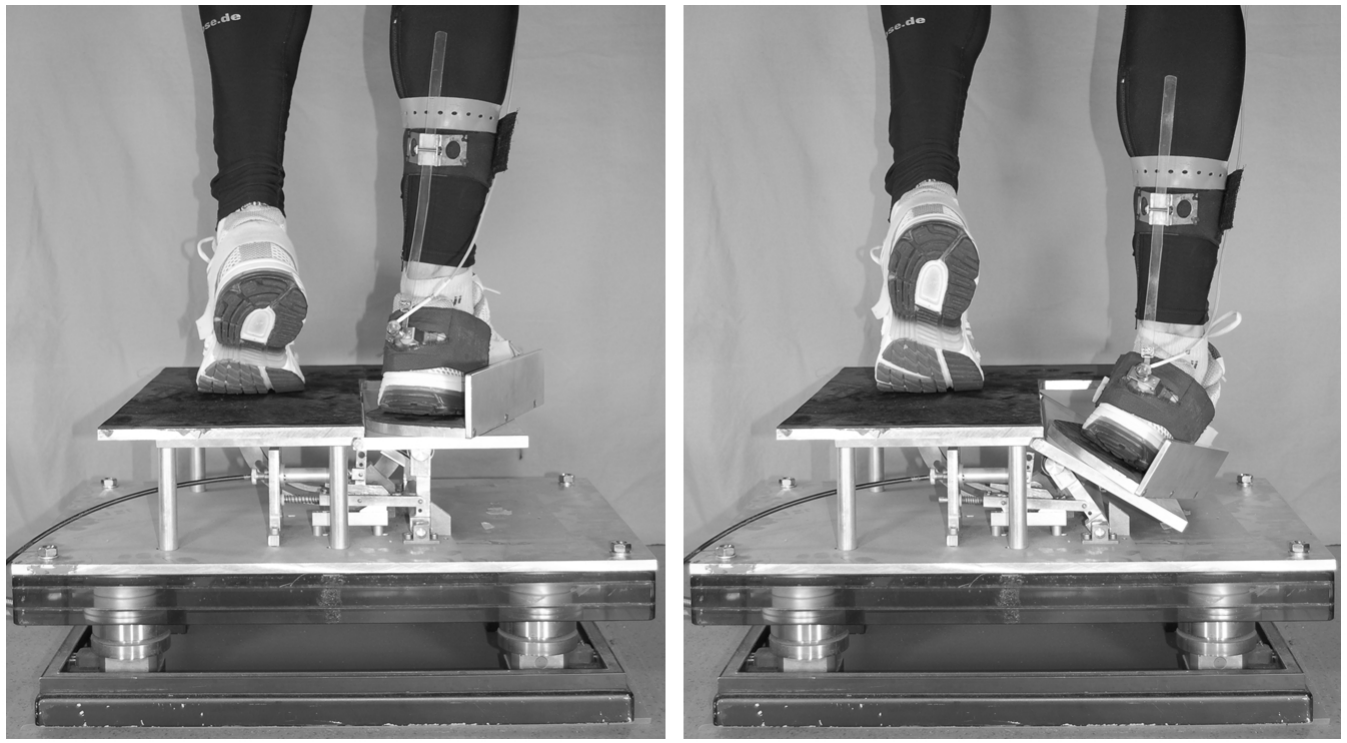
Laboratory setting of the sudden ankle supination.

A custom made supination platform mounted on a force plate (Kistler 9281B, Winterthur, CH) was used (Fig 2) to monitor experimentally foot behaviour and muscle reaction time (EMG) during a sudden ankle turn. The functional portion of the platform was a movable section which could be dropped to an angle of 26° sideways. For protection, the tiltable part of the platform was constructed with a boundary to prevent a sideslip of the foot. The rotation axis of the trapdoor was just medial of the weight-bearing foot, and only the toes of the other foot were placed in contact to the trapdoor to maintain balance. According to Podzielny and Hennig [27], the weight-bearing foot was oriented laterally so that the angle of the foot relative to the platform axis was 13°. Therefore, the platform release enabled a forceful inversion and plantarflexion of the foot (Fig 2). From the force platform data, peak vertical ground reaction force after touchdown (F_Max_) and the time from platform release to F_Max_ (t_FMax_) were evaluated.

The supination angle was measured by the change of Achilles tendon angle with reference to the Achilles tendon angle during bipedal standing by using an electrogoniometer. A lightweight half-circular metal construction with a conductive plastic potentiometer (MP 10, Megatron, Munich, Germany) was attached to the heel counter of a sports shoe. Its axis of rotation was positioned at the approximate height of the subtalar joint. The movable part of the goniometer was fixed in a guiding metal rail at the lower leg in alignment with the Achilles tendon orientation. All subjects wore the same shoe model (Deichmann, Victory Performance), provided in EU sizes 41–46. Thereby, we could measure the shoe counter movement relative to the shank in the frontal plane, but were unable to differentiate between the shoe and the foot movement with this setup. However, Stacoff et al. [28] and Milani & Hennig [29] showed that foot and shoe show very similar angular movement patterns relative to the shank. Therefore, we assumed that the movement of the shoe counter represents the heel foot motion inside of the shoe sufficiently well. To avoid measurement errors due to differences in the goniometer application between pre-test and post-test, we favoured rearfoot motion analysis in shod condition. To compare rearfoot motion data, the goniometer method shows high correlations to motion analysis systems [30]. Maximum supination angle (Sup_Max_) and maximum supination velocity (SDGN) were calculated from the goniometer values.

Parallel to the measurements of rearfoot motion, the muscular reactions of tibialis anterior (TA), peronaeus longus (PL) and vastus medialis (VM) were recorded by surface EMG electrodes (Delsys, Boston, USA). All electrode placements were performed according to the SENIAM recommendations [31] by the same investigator, and their correct placement was checked by manual tests and voluntary contractions. The reference electrode was placed over the malleolus lateralis fibulae. In random order, five measurements each were performed while standing on both legs. Only when the EMG signals showed baseline activity, the tilting platform was released. To eliminate auditory cues to the platform release, subjects wore a headphone. The raw EMG signal was A/D converted, sampled at a frequency of 4000-Hz and 12-bit resolution. A band pass filter (built into the amplifier) of 20–450 Hz was applied to the raw electrical input of the surface electrodes before full-wave rectification. EMG onset times were manually determined for each trial because visual onset determination was found to be more robust compared to algorithmic methods. EMG onset was identified when the EMG response showed a steep and marked activity increase that was at least higher than two standard deviations of the baseline activity being measured 1 second before the start of the tilting. The time span from the moment triggering the release of the tilting platform to the first EMG response was defined as the muscle reaction time or muscle onset time. Integrated EMG (IEMG) of PL, TA and VM was evaluated for the first 60ms after EMG onset when voluntary muscle contraction sets in [32]. To compare the amount of activity between pre- and post-test, IEMG was separately normalised for each tested muscle. Reference contraction for PL was the maximum voluntary pronation during the strength measurement by using the SPSM. IEMG of TA was normalised by maximum voluntary dorsiflexion by using the dorsiflexor strength training machine. For these tests, the subjects were tested in a seated position (approximately 90° in hip and knee joint) without movement of their knee and hip joint. The hip was fixed with a belt, and the subjects were instructed to cross their arms during the testing. For VM normalisation, the subjects sat on a stool with a rectangular position of hip and knee joint, leaning against the wall with their back. Then, the subjects were instructed to strongly push their back against the wall and to hold this position when the stool was removed. Subsequently, they were asked to lift one foot from the floor. In this position, when the quadriceps muscle of the stance leg was nearly complete activated, the respective reference VM muscle activity was recorded. All EMG data were analysed blindly by the same investigator.

#### Muscle volume measurements

Magnetic resonance imaging (MRI) was used to analyse changes in muscle volume induced by shank muscle strength training in 9 randomly chosen test persons of the experimental group. Before and after the intervention, both shanks were scanned by using a 3-Tesla MRI scanner system (Philips Intera Achieva, Eindhoven, NL).

Reproducible positioning of the test persons in the pre- and post-test was provided by the standard Philips foot plate: At rest, the knees were slightly flexed and placed on a cushion. The feet were plantar flexed at nearly 10° (where 0° is with the sole of the foot at right angle to the lower-leg axis) and fixed with belts around the feet’s dorsum. The hip joints were slightly externally rotated. All images were taken after 15 minutes of rest to allow fluid shifts that occur when changing from upright to supine position [33].

Across a length of 360mm from the intracondylar notch to distal, 75 T1-weighted transaxial images of the lower leg were obtained using a fast inversion recovery pulse sequence at a rejection time (TR) of 1232.5ms and an echo time (TE) of 10ms in transverse planes. The transverse axial slice thickness was 4mm with slice gaps of 8mm and a field of view (FOV) of 360mm. The anatomical cross sectional area (ACSA) of every slice was manually digitised by the graphic tools of the standard MRI evaluation software (View Forum, release 2.5.3.3, Philips, Eindhoven, NL). On the transverse view, the margins of the corresponding muscles were identified with a cursor on the computer screen. Eleven slices of each muscle or muscle group, respectively, were evaluated.

After identifying the largest ACSA, the next five distal and proximal slices were analysed. This procedure was repeated for each muscle. The muscle volume was determined by the ACSAs multiplied by the summarised slice thickness and added to the volumes of the slice gaps. The volume of each slice gap was calculated by a multiplication of the slice gap with the arithmetic average of the two, distally and proximally, enclosing ACSAs. The muscle volumes of the following muscles and muscle groups, respectively, were determined: dorsiflexors (DF), peroneal muscles (PER), gastrocnemius (GAS), soleus (SOL), and the deep supinators (SUP, i.e. tibialis posterior, flexor hallucis longus, flexor digitorum longus). The same tester (AG) digitised blindly each ACSA of each of the muscles on two different days. Intraclass correlation coefficients between the two sessions for the analysed muscles ranged from 0.81 to 0.93. The overall intraclass correlation coefficient was 0.88. For interference statistical analysis, the average muscle volumes of day 1 and 2 were used. Note that the indicated muscle volumes are not equal to the whole volumes of the analysed muscles/muscle groups, but they are valid representatives of their morphology. Therefore, changes of these values are indicators for the morphological strength training adaptations.

### Strength Training Programme

Over a period of 10 weeks, the subjects performed 3 weekly training sessions. In each training session, single-set high-resistance shank muscle strength training was performed until task failure within eight to ten repetitions. An intensity procedure was applied to achieve an even higher amount of exposure (Table 1). After task failure, the target load was reduced by 10%, and the subjects were instructed to perform additional repetitions until volitional exhaustion. The same load reduction procedure was repeated in a third set. The training load and the number of repetitions were documented during each training session. The subjects were instructed to increase their training load progressively in order to stimulate the target muscles with a high intensity during each session. All training sessions were directly supervised and documented by an experienced personal trainer. The personal trainer was also an assessor of the study who briefed the subjects to perform each exercise until volitional exhaustion.

**Table 1:** Three-step intensity procedure during single-set strength training.

Step	Load	Repetitions
1	Target load	8–10 repetitions until task failure
2	Target load minus 10%	Additional repetitions until task failure
3	Target load minus 20%	Additional repetitions until task failure

The subjects conducted ST with the right leg by using a custom-made training machine (Fig 1), the left leg was trained by TT at traditional training machines (“Calftrainer PSC-43-PL”, Powerline, USA; dorsiflexor machine “B8”, Kieser Training, Switzerland).

### Data Analysis

Statistical analyses were performed using SPSS version 22.0 (SPSS Inc., Chicago, Illinois, USA). The level of significance was set at α = 5%. The measurement parameters were averaged for each subject before further statistical treatment. Confirmation that all dependent variables were normally distributed was assessed using repeated Kolmogorov-Smirnov tests. As muscle strength, muscle volume, muscle activity and biomechanical (goniometer, force plate) measurements test different constructs, the dependent variables were grouped into 5 main groups: 1 –muscle strength; 2 –muscle volume; 3 –reaction times (stretch reflex);- 4 –voluntary activity; and 5 –mechanical stability. Subsequently, 5 separate 2x2 mixed-model repeated measures multivariate analyses of variance (MANOVA) designs [time (pre- vs posttest x group (ST vs. TT)] were performed. Significant multivariate tests were followed by univariate ANOVAs with subsequent Bonferroni corrections. In addition, the classification of effect sizes was determined by calculating partial eta square (η^2^_p_).

## Results

The descriptive results of the analysed parameters are presented in Tables 2 and 3 according to the 5 separate MANOVAs.

**Table 2:** Training-induced changes of supination behavior, peak ground reaction forces and muscle activity during sudden ankle supinations after talocrural plantar-/dorsiflexor training (TT) and subtalar pronator/supinator training (ST).

		TT		ST	
		Pre-training	Post-training	Pre-training	Post-training
**Mechanical stability** ^**A**^					
	**Sup**_**Max**_ **(°)**	26.5 ± 0.7	26.6 ± 0.8	26.7 ± 0.44	26.6 ± 1
	**SDGN (°/s)** ^**a**^	604.2 ± 141.5	532.8 ± 91.8	562.2 ± 149.2	502.2 ± 123.7
	**F**_**Max**_ **(bw)**	1.61 ± 0.16	1.57 ± 0.15	1.65 ± 0.15	1.6 ± 0.12
	**t**_**FMax**_ **(ms)**	130.6 ± 16.3	135.8 ± 17.6	132.8 ± 22	137.4 ± 13.2
**Reaction times** ^**A**^					
	**RT**_**PL**_ **(ms)** ^**a**^	59.3 ± 8.6	56.0 ± 7.6	61.7 ± 4.7	54.5 ± 7.3
	**RT**_**TA**_ **(ms)**	62.4 ± 10.0	58.5 ± 10.3	60.7 ± 11.4	59 ± 11
	**RT**_**VM**_ **(ms)**	60.9 ± 9.3	60.4 ± 10.2	60.9 ± 10.2	63 ± 9.8
**Voluntary activation**					
	**IEMG**_**PL**_ **(% MVC)**	36.0 ± 16.6	43 ± 13.8	38.1 ± 14.7	36.8 ± 9.9
	**IEMG**_**TA**_ **(% MVC)**	32.0 ± 12.3	33.8 ± 10.6	29.2 ± 13.3	30.8 ± 11
	**IEMG**_**VM**_ **(% MVC)**	19.3 ± 8.1	20.6 ± 8.5	22.8 ± 12.9	20.6 ± 12
	**TIEMG**_**PL**_ **(ms)**	118.9 ± 17.0	116.0 ± 17.5	117.1 ± 11.9	118.5 ± 10.8
	**TIEMG**_**TA**_ **(ms)**	128.3 ± 19.7	120.9 ± 15	121.4 ± 11.7	121.9 ± 13.4
	**TIEMG**_**VM**_ **(ms)**	122.4 ± 24.5	118 ± 19.3	128.1 ± 30.6	126.9 ± 24.7

^A^, significant main effect (time) in MANOVA;

^a^, significant main effect (time) in ANOVA.

Parameters: Sup_Max_ = maximum supination angle; SDGN = maximum supination velocity; F_Max_ = peak vertical force after touchdown; t_FMax_ = time from platform release to peak vertical force; RT_PL_ = reaction time of peroneus longus; RT_TA_ = reaction time of tibialis anterior; RT_VM_ = reaction time of vastus medialis; IEMG_PL_ = integrated EMG of peroneus longus; IEMG_TA_ = integrated EMG of tibialis anterior; IEMG_VM_ = integrated EMG of vastus medialis; TIEMG_PL_ = time to voluntary EMG onset of peroneus longus; TIEMG_TA_ = time to voluntary EMG onset of tibialis anterior; TIEMG_VM_ = time to voluntary EMG onset of vastus medialis.

**Table 3:** Training-induced changes of muscle strength and volume after talocrural plantar-/dorsiflexor training (TT) and subtalar functional pronator/supinator training (ST).

		TT		ST	
		Pre-training	Post-training	Pre-training	Post-training
**Muscle strength** ^**A**^ ^**,**^ ^**B**^ **in Nm**					
	**Pronator MVIC** ^**a**^ ^**,**^ ^**b**^	22.0 ± 5.9	23.7 ± 5.7	22.1 ± 5.3	25.3 ± 5.6
	**Supinator MVIC** ^**a**^ ^**,**^ ^**b**^	20.9 ± 4.5	23.4 ± 4.8	20.5 ± 5.1	25.4 ± 4.7
**Muscle volume** ^**A**^ **in cm** ^**3**^					
	**dorsiflexors (DF)** ^**a**^	61.1 ± 9.9	63.8 ± 12.2	63.9 ± 9.0	66.2 ± 11.7
	**Peroneal muscles (PER)** ^**a**^	31.8 ± 7.6	33.3 ± 8.9	33.2 ± 5.6	35.2 ± 7.6
	**gastrocnemius (GAS)**	130.7 ± 23.1	141.9 ± 28.9	143.1 ± 29.2	151.9 ± 38.0
	**soleus (SOL)**	111.1 ± 21.4	114.6 ± 22.7	116.7 ± 23.1	118.1 ± 28.6
	**supiators (SUP)** ^**a**^	80.8 ± 13.0	82.5 ± 12.5	78.3 ± 12.0	83.5 ± 14.3

MVIC: Maximum voluntary isometric contraction.

^A^, significant main effect (time) in MANOVA;

^B^, significant interaction (group x time) in MANOVA;

^a^, significant main effect (time) in ANOVA;

^b^, significant interaction (group x time) in ANOVA.

Compared to TT, ST leads to significantly higher pronator (14% vs. 8%) and supinator strength (25% vs. 12%). The ‘muscle strength’ MANOVA produced a significant main effect for time [Wilk’s Lambda = 0.168; F (2,41) = 101.6; P<0.0001; η^2^_p_ = 0.832] and a significant time x group interaction [Wilk’s Lambda = 0.641; F (2,41) = 11.5; P<0.0001; η^2^_p_ = 0.359]. Univariate analyses revealed significant main effects for time for pronator [F (1,42) = 132.42; P<0.0001; η^2^_p_ = 0.759] and supinator strength [F (1,42) = 140.77; P<0.0001; η^2^_p_ = 0.770] as well as significant time x group interactions for both pronator strength [F (1,42) = 12.97; P<0.0001; η^2^p = 0.236] and supinator strength [F (1,42) = 17.69; P<0.0001; η^2^_p_ = 0.296]. There was no main effect for group.

The MANOVA for ‘muscle volume’ revealed a significant multivariate main effect for time [Wilk’s Lambda = 0.324; F (5,12) = 5.01; P = 0.01; η^2^_p_ = 0.677]. The following univariate ANOVAs showed significant main effects for time for DF [F (1,16) = 9.67; P = 0.007; η^2^_p_ = 0.377], PER [F (1,16) = 10.15; P = 0.006; η^2^_p_ = 0.388], SUP [F (1,16) = 24.79; P = 0.0001; η^2^_p_ = 0.608] and a trend to main effect for time for GAS [F (1,16) = 8.04; P = 0.012; η^2^_p_ = 0.335]. SUP showed a trend to a time x group interaction [F (1,16) = 6.72; P = 0.02; η^2^_p_ = 0.296]. There was no group x time interaction or main effect for group.

**Fig 3:**
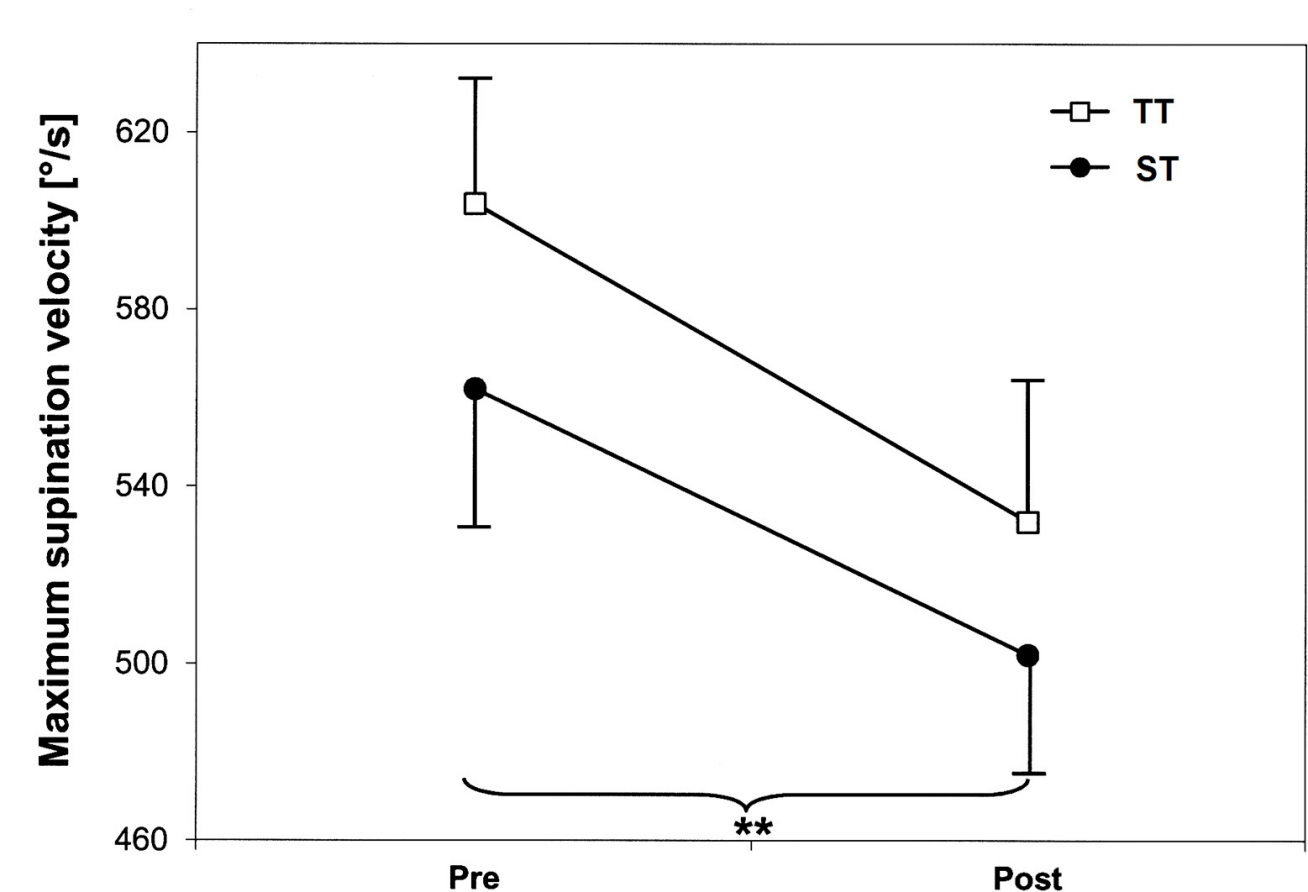
Strength training-induced changes in maximum supination velocity (means and standard errors). TT = plantar-/dorsiflexor training; ST = functional subtalar pronator/supinator training; ** indicates significant main effect (time) (p<0.01).

During sudden inversions, shank muscle strength training leads to reductions in peak vertical ground reaction force of 3% (ST) and 2.5% (TT) (Table 2). Supination velocity was decreased by 11% and 12% after ST and TT, respectively (Fig 3). The MANOVA for ‘mechanical stability’ detected a significant main effect for time [Wilk’s Lambda = 0.708; F (4,39) = 4.02; P = 0.008; η^2^_p_ = 0.292]. The subsequent univariate ANOVAs showed a significant time effect for supination velocity [F (1,42) = 9.43; P = 0.004; η^2^_p_ = 0.183]. Time x group interactions were not found.

**Fig 4:**
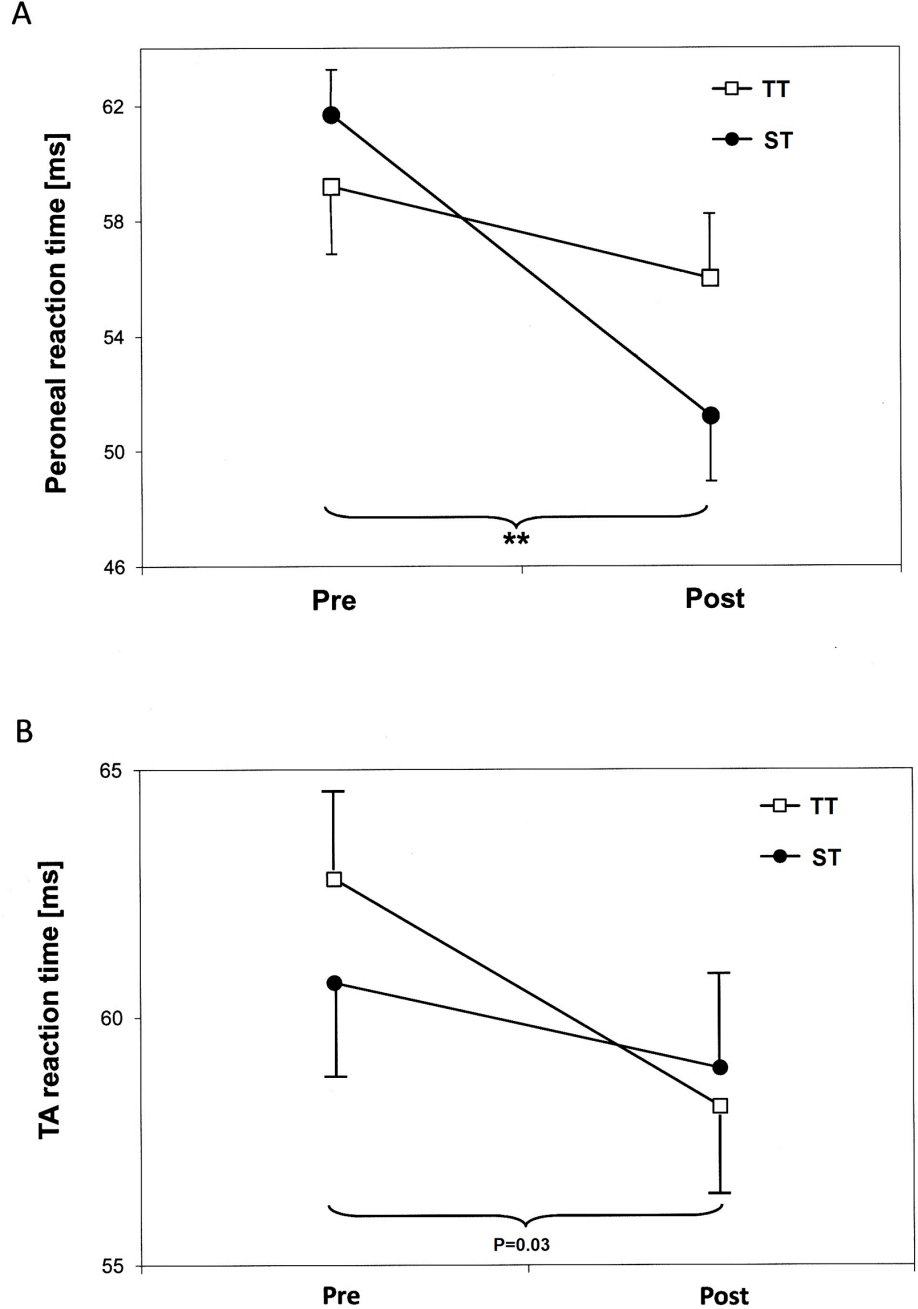
Strength training-induced changes in muscular reaction time of peroneus longus (A) and anterior tibial (B) muscles (means and standard errors). TT = plantar-/dorsiflexor training; ST = functional subtalar pronator/supinator training; ** indicates significant main effect (time) (p<0.01).

Muscular reaction time on a sudden ankle turn was faster in PL by 12% and 6% after ST and TT, respectively (Fig 4). The MANOVA for ‘reaction times’ revealed a significant multivariate effect for time [Wilk’s Lambda = 0.641; F (3,39) = 7.286; P = 0.001; η^2^_p_ = 0.359]. The following univariate ANOVAs showed a significant main effect for time for PL [F (1,41) = 22.91; P = 0.0001; η^2^_p_ = 0.358] and a trend towards a main effect for time for TA [F (1,41) = 5.26; P = 0.027; η^2^_p_ = 0.114]. Time x group interactions were not found.

The MANOVA for the ‘voluntary muscle activation’ did not show any significant effects (Table 2).

## Discussion

The purpose of this study was to investigate the morphological and biomechanical effects of two machine-based shank muscle training methods: a) Subtalar pronator and supinator strength training by application of resistance around the subtalar joint axis (ST), and b) talocrural plantar- and dorsiflexor strength training around the talocrural joint axis (TT). It was hypothesised that the peroneal muscles would demonstrate greater increases in muscle strength and thickness with ST over TT, which may result in enhanced neuromuscular control during a sudden inversion.

The findings of the study show evidence of increased lateral stability after shank muscle strength training when a sudden inversion is induced, but our hypothesis of ST-specific effects on the peroneal muscles cannot be confirmed. In fact, the increase in pronator and supinator MVIC is significantly dependent on the specificity in training in favour of ST, but the morphological and biomechanical effects do not reveal significant interactions between ST and TT.

The absence of time x training interactions in biomechanical outcome measures can be explained by the muscle volume findings. Both ST and TT result in significant muscle volume increase in PL and TA, but without significant time x training interactions. This finding is also reflected in the amount of muscle activity (integrated EMG) when pronator and dorsiflexor normalisation procedures were performed. PL shows highest activity (100%) during measurement of pronation MVC, but PL is also highly active when the foot is in maximum voluntary dorsiflexion (73%), and, even higher, in single leg heel-rise (87%). When the foot is dorsiflexed by the muscle action of TA, the foot is also moved into relative supination because the tension of TA pulls on the medial side of the foot. Subsequently, coactivating PL is necessary to counteract the supination torque when an isolated dorsiflexion of the foot is requested. During heel-rises, PL is activated as plantarflexor. When an isolated plantarflexion is performed, PL is required to work against the supination torque induced by triceps surae. TA is highly active (80%) when the foot is pronated. Pronation is a three-dimensional movement of the foot around the oblique subtalar joint axis, and consists of three components: eversion and abduction of the foot, combined with relative dorsiflexion [34]. As a consequence, the more the foot is pronated, the more TA contributes to the resulting pronation torque. Therefore, both ST and TT exercises overloaded PL and TA so that no time x group interactions were found in muscle volume changes. However, ST-dependent strength increase in pronation MVIC can be traced back to higher neural drive of PL as the subjects of the ST group were more adapted to the specific subtalar pronator exercises which were also tasked in the MVIC test.

In contrast to TA and PL, the ST-specific enhancement in supination MVC goes with obvious muscle volume increases of the deep plantarflexors which showed a strong time x group interaction trend (P = 0.02). In a recent experiment it was observed that the pronation behaviour of the foot during ground contact in overground running is remarkably better muscularly controlled after ST as compared to TT [35].

### Mechanical Stability

**Fig 5:**
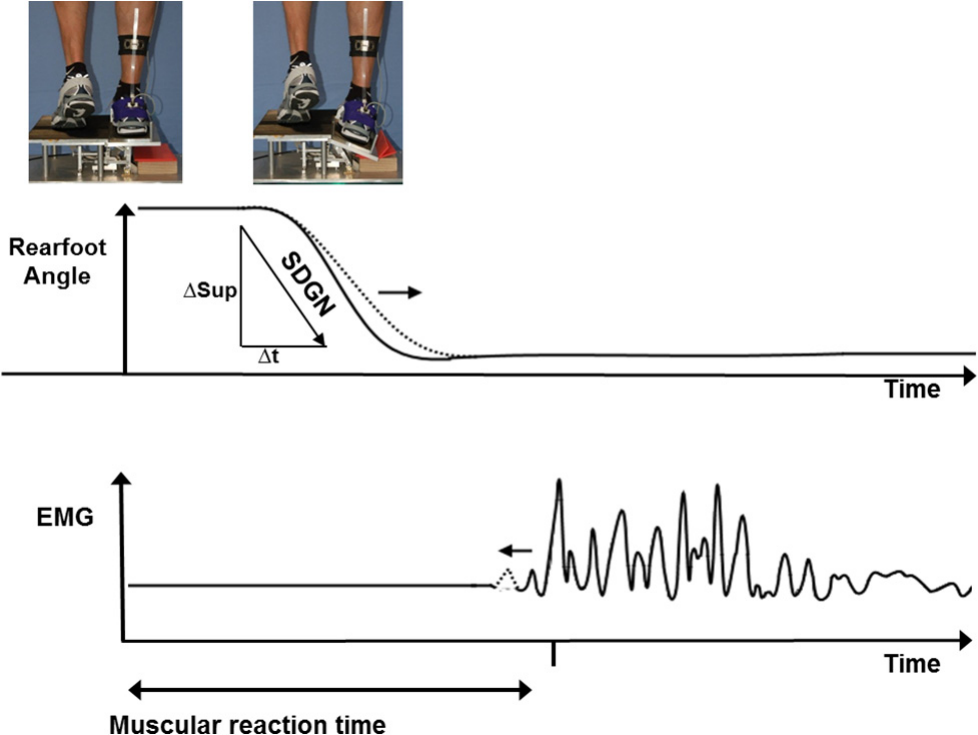
Examplary biomechanical changes (rearfoot motion (top), vertical forces (middle), EMG (bottom)) after strength training (dotted line) when compared to pre-testing. The arrows indicate a significant reduction of supination velocity (SDGN, rate of decline in the goniometer signal during free fall) and a significantly shorter muscular reaction time after releasing the tilting platform.

The main finding of the present study is that systematic machine-based high-resistance training of the shank muscles leads to increasing stability of the foot when a sudden ankle inversion is induced. After both ST and TT, the stabilising effects become obvious within the first 75ms after the platform was released (Fig 5). The subjects did not know the time of sudden foot supination caused by the trigger release of the tilting platform. Therefore, anticipatory activity of the antagonistic muscles can be excluded, so the foot was not voluntarily controlled during this period. Thus, it can be assumed that the main strength training effect is a rising mechanical joint stability due to strength and muscle volume increase of PL and the dorsiflexors caused by proliferation of myofibrils [36]. If we compare the function of a muscle with an adjustable spring whose coefficient of elasticity is modulated by the number of springs arranged in parallel, we will be able to increase the stiffness of the spring configuration by muscle hypertrophy. Following Hooke’s law, a certain force would only lead to half displacement if the number of springs in parallel was doubled. Therefore, a strength training-induced increase of the number of titin-myosin-units as well as a higher potential of resting cross bridges in terms of short-range elastic component [37] are attended by higher resistance of the joint complex against an external force [38]. These effects might stiffen the ankle joint complex mechanically and act against a supination movement under a loading condition. Due to the oblique tilting axis of the platform used in our experiment, which induced a combined plantarflexion and supination movement of the foot, the amount of supination velocity was dependent on the muscle stiffness of both the pronators and dorsiflexors. The results of our study support the stiffness hypothesis, as we found a significant main effect of time on both muscle volume of DF and PER and supination velocity, but no time x group interactions.

It may be speculated that reduced reaction times in TA and PL are a consequence of faster mechanical transfer of tendon stretch caused by the higher stiffness of the muscle-tendon complex. Fellows & Thilmann [39] found a highly linear relationship between muscle length and stretch reflex latency in triceps surae. The more the muscle is stretched, the quicker the muscle reaction time will be. For slacker tendons, the initial muscular reaction time increases. In contrast, Blackburn et al. [40] found that a moderate level of pretension at 30% of MVIC likely eliminates series elastic slack. In the present experiment, the tilting platform was released when the foot was in neutral position and PL only showed baseline activity. However, during sudden ankle supination the amount of musculotendinous stiffness of TA and PL was not measured so that the mechanical influence of training-induced changes in PL and TA on the depolarisation of the muscle spindle receptors is still speculative.

### Neuronal effects

Discussing the reduced reaction times of PL and TA, training-induced changes in neuronal organisation should also be considered because it is believed that strength gains during the first three to five weeks of strength training are primarily due to neural factors [36], [41]. For example, strength training has been reported to influence motor unit recruitment, selective activation of agonist muscles and their motor units, and antagonist coactivation. Especially inhibitory presynaptic influences seem to modulate the synaptic plasticity of the motoneuron pool [42]. Furthermore, faster nerve conduction velocities during maximum voluntary contraction were found in sprinters as compared to endurance runners [43]. These findings could be attributed to exercise-dependent hypertrophy of alpha motoneurons which were observed in animal studies [44], [45]. Nevertheless, it has to be stated that the findings on morphological adaptations of alpha motoneurons are inconsistent [46]. It is also unclear whether longer-term neuronal effects extend to muscle proprioceptors. Docherty et al. [12] discuss enhanced receptor sensitivity of the muscle spindle after strength training which was observed after voluntary muscle contraction [47]. Subsequently, faster muscle spindle stimulation could also reduce the muscular reaction time. Nevertheless, it must be concluded that the neuronal effects are not sufficiently understood to fully explain our findings of reduced reaction times of PL and TA. In the present study, EMG recordings were applied to identify the potential effects of ST and TT on motor unit recruitment and firing rate which are reflected in IEMG. However, changes in voluntary muscle activation in the period of 60 ms, after the first reflex response occurred, are not present. Although neural factors might influence the reaction times, we have to mention that, to our understanding, the mechanical effects of shank muscle strength training on the lateral stability are predominant, as discussed earlier.

### Limitations of the study

The strength testing and ST intervention were conducted by using a custom-made subtalar pronator and supinator machine. As described in the methods section, training and testing was limited to a seated non-weight-bearing position due to the specific apparatus.

Furthermore, ST exercising was possible only for the right leg, and the study design differs from a crossover study. Another study limitation is the potential influence of the leg dominance on the differential effects of the training programmes, i.e. the influence of the dominant versus the non-dominant limb. Muscular reaction times of VM, TA and PL were identified manually because it appeared more robust compared to algorithmic methods. However, the reliability of manual determination of the EMG onset was not tested and thus has to be mentioned as a limitation of the study. Furthermore, the reliability of the VM normalization procedure, which was not an MVIC, was not tested.

### Shank muscle strength training for the prevention of ankle sprains

The findings of reduced supination velocities reveal that both shank muscle strength training methods, ST and TT, lead to enhanced mechanical ankle stability. We applied two strength training machines and one seated heel-rise apparatus to strengthen the target muscles in our study. While other studies were unable to find significant stabilising effects after strength training interventions with elastic rubbers [12], [13], [14], it can be assumed that systematic effects are dependent on the appropriate application of resistance. For achieving muscle volume increase, which is essential for stronger bracing of the foot-shank complex, it is required to load the target muscles above an individual myofibril threshold. This requirement is not necessarily given in strength training exercises with elastic rubbers, but it obviously is in machine-based resistance regimens as applied in our intervention.

The results deliver evidence that machine-based shank muscle strength training is beneficial for enhancing ankle stability in a standing position when a sudden inversion is induced. The pathophysiology of the most frequent sports injury, the lateral ankle sprain, is typically a landing manoeuvre, most frequently in team sports. As the antigravity triceps surae is pre-innervated to dampen the impact of landing, the foot is in a plantarflexed and supinated position. If the foot unfortunately lands in an disadvantageous position (i.e. higher degree of supination or plantarflexion) or on an irregular surface, such as the foot of another player, the ground reaction force moment arm about the subtalar joint will increase [11], [48], [49]. The inability to accurately position the foot prior touchdown is seen as the most important factor which contributes to increased ankle sprain occurrence [5].

As the reduced supination velocities indicate, the strength training effects are already present during free-fall. According to the mechanism of an ankle injury, the increase in mechanical stability is very important as feed-back control is not effective due to the motor response and the electromechanical delay of the stabilising muscles [7]. The increasing stiffness of the peroneal and dorsiflexor muscles will probably influence subsequent rearfoot motion during load-bearing at contact. If less inversion on the rearfoot is present at initial contact, the ground reaction force moment arm about the subtalar joint will be reduced. Therefore, the moment will force the foot into less inversion. This bracing function may be more important than its function as force bypass to the ligaments. Therefore, the mechanical effects of strength training on foot stability are important for preventive training programmes [17], even more so to prevent patients from a recurrent ankle sprain as strength deficits were observed in subjects with unstable ankles [50], [51], [52].

The strength-training effects on the reaction times of TA and PL have to be considered according to the dynamic response to sudden ankle-foot supination. As shown by Konradsen et al. [7] in an experimental setting, the reaction pattern is too slow to prevent lateral ligament overloading. The same authors assume that only anticipated preinversion muscle activity (feed-forward control), the strength of the static stabilizers, or the protection of an external support can prevent an inversion injury. As mentioned earlier, the reduced reaction times of TA and PL can also result from their increased musculotendinous stiffness after ST and TT.

Muscle strength increase is also an influential factor to improve proprioception. Proprioceptive training is applied to influence the feed-forward control in terms of pre-activating the laterally stabilising muscles. Not only the increase in muscle stiffness of the pronators and dorsiflexors would contribute to appropriate foot positioning prior touchdown, but also an adequate amount of strength capacity is necessary so that enhanced proprioception can come into effect. It can be assumed that machine-based pronator and dorsiflexor strength training combined with proprioceptive exercises would result in even higher foot stability.

Finally, it has to be mentioned that the strength training applied in the present study was performed by healthy male sports students. Contraindications like pain and discomfort may be considered when strength training is applied in early rehabilitation after an ankle trauma, especially when the ankle and subtalar joint is strengthened across the full range of motion. In a pilot study, pain-free patients with instable ankles, who suffered from recurrent ankle sprains, felt comfortable while training the pronators and dorsiflexors by using the machines. Consequently, future clinical studies are recommended to investigate the preventive potential of machine-based shank muscle strength training in patients with unstable ankles. It is hypothesised that the stabilising effects would become even more obvious in these patients than in healthy subjects.
